# New Books

**Published:** 2007-11

Against Bioethics

Jonathan Baron

Cambridge, MA:MIT Press, 2007. 248 pp. ISBN: 978-0-262-52478-0, $16

An Environmental History of Latin America

Shawn William Miller

Cambridge, UK:Cambridge University Press, 2007. 272 pp. ISBN: 978-0-521-61298-2, $26.54

Analysis of Global Change Assessments: Lessons Learned

Committee on Analysis of Global Change Assessments, National Research Council

Washington, DC:National Academies Press, 2007. 196 pp. ISBN: 978-0-309-10485-8, $42.75

Biodiversity under Threat

R.M. Harrison, R.E. Hester, eds.

New York:Springer, 2008. 214 pp. ISBN: 978-0-85404-251-7, $99

Cool It: The Skeptical Environmentalist’s Guide to Global Warming

Bjørn Lomborg

New York:Knopf Publishing, 2007. 272 pp. ISBN: 978-0-307-26692-7, $21

Current Controversies in the Biological Sciences

Karen F. Greif, Jon F. Merz

Cambridge, MA:MIT Press, 2007. 399 pp. ISBN: 978-0-262-07280-9, $62

Energy Futures and Urban Air Pollution: Challenges for China and the United States

Committee on Energy Futures and Air Pollution in Urban China and the United States

Washington, DC:National Academies Press, 2007. 420 pp. ISBN: 978-0-309-11140-4, $79

Environmental Policy Analyses

Peter Knoepfel

New York:Springer, 2007. 506 pp. ISBN: 978-3-540-73148-1, $209

Fundamentals of Air Pollution, 4th ed.

Daniel Vallero

Burlington, MA:Elsevier, 2007. 968 pp. ISBN: 978-0-12-373615-4, $89.95

Human Impacts on Weather and Climates, 2nd ed.

William R. Cotton, Roger A. Pielke, Sr.

Cambridge, UK:Cambridge University Press, 2007. 320 pp. ISBN: 978-0-521-60056-9, $61.28

Hot House: Global Climate Change and the Human Condition

Robert G. Strom

New York:Springer, 2007. 306 pp. ISBN: 978-0-387-34179-8, $27.50

Methods for Computational Gene Prediction

William H. Majoros

Cambridge, UK:Cambridge University Press, 2007. 448 pp. ISBN: 978-0-521-87751-0, $143.04

Microarrays

Jang B. Rampal, ed.

New York:Springer, 2007. 452 pp. ISBN: 978-1-58829-944-4, $125

Nanotechnologies, Hazards and Resource Efficiency

M. Steinfeldt, A.V. Gleich, U. Petschow, R. Haum

New York:Springer, 2007. 271 pp. ISBN: 978-3-540-73882-4, $139

Persistent Organic Pollutants in Asia

An Li, Shinsuke Tanabe, Guibin Jiang, John Giesy, Paul Lam, eds.

Burlington, MA:Elsevier, 2007. 842 pp. ISBN: 978-0-08-045132-9, $185

Principles of Environmental Physics, 3rd ed.

John Monteith, Mike Unsworth

Burlington, MA:Elsevier, 2007. 440 pp. ISBN: 978-0-12-505103-3, $49.95

The Blue Death: Disease, Disaster, and the Water We Drink

Robert D. Morris

New York:HarperCollins, 2007. 320 pp. ISBN: 978-0-06-073089-5, $24.95

